# Integration of unpaired single cell omics data by deep transfer graph convolutional network

**DOI:** 10.1371/journal.pcbi.1012625

**Published:** 2025-01-16

**Authors:** Yulong Kan, Yunjing Qi, Zhongxiao Zhang, Xikeng Liang, Weihao Wang, Shuilin Jin

**Affiliations:** School of Mathematics/Harbin Institute of Technology, Harbin, China; University of California Irvine, UNITED STATES OF AMERICA

## Abstract

The rapid advance of large-scale atlas-level single cell RNA sequences and single-cell chromatin accessibility data provide extraordinary avenues to broad and deep insight into complex biological mechanism. Leveraging the datasets and transfering labels from scRNA-seq to scATAC-seq will empower the exploration of single-cell omics data. However, the current label transfer methods have limited performance, largely due to the lower capable of preserving fine-grained cell populations and intrinsic or extrinsic heterogeneity between datasets. Here, we present a robust deep transfer model based graph convolutional network, scTGCN, which achieves versatile performance in preserving biological variation, while achieving integration hundreds of thousands cells in minutes with low memory consumption. We show that scTGCN is powerful to the integration of mouse atlas data and multimodal data generated from APSA-seq and CITE-seq. Thus, scTGCN shows high label transfer accuracy and effectively knowledge transfer across different modalities.

## Author summary

Single-cell omics technologies have significantly advanced our ability to study biological systems at an unprecedented resolution and scale. However, integrating the multimodal single-cell data that emerges from these technologies—such as finding cell-to-cell correspondences, gene-peak relationships, and conducting cell pseudotime analysis—remains a complex challenge. Alongside the advancements in single-cell technologies, deep learning (DL), a revolutionary development in artificial intelligence, has transformed our capacity to analyze large-scale data through sophisticated neural network architectures. The efficacy of DL was recently showcased by AlphaFold2’s success in predicting protein structures. In response to these challenges, we propose a flexible deep transfer learning model for the comprehensive analysis of unpaired single-cell multiomics data. Our method not only integrates scRNA-seq and scATAC-seq data but also refines and provides new annotations through this integrated analysis.

## Introduction

Single-cell genomics give us new perspectives to understand the genetics in the cellular level. With the rapid development of methods for single cell RNA sequences such as 10X Chromium (PBMC), Smart-seq2 [1], SNARE-seq [2] and SHARE-seq [3]. Technologies for measure other modalities, single-cell chromatin accessibility sequence [4], proteomics [5, 6], spatial transcriptomics [7] and metabolomics [8] have been developed. However, each technology only reveal a particular aspect of cellular state and identity. They both have unique strengths and weaknesses.

In particular, scATAC-seq is a strong indicator of functional DNA sequence which can be used to identify cell subpopulations with different chromatin accessibility profiles. Nevertheless, scATAC-seq coupled with the sparsity of signals due to low detection efficiency and cell-type identify accuracy present a huge computational challenge. In contrast, large amounts of well-annotated scRNA-seq data have been curated [9], catalyzing us to transfer cell type information from scRNA-seq to scATAC-seq.

Integration of single-cell multi omics data is still a great challenge due to the heterogeneity across multiple datasets, including inherent highly sparse and imcompatible dimension between scRNA-seq and scATAC-seq data. A large number of methods have been developed for scRNA-seq data integration. Such as scmp [10], scAlign [11], DCA [12], scVI [13]. And many methods have been developed for scATAC-seq data including chromVAR [14], cisTopic [15], Scasat [16] and SnapATAC [17]. The methods were proposed for integrating paired and unpaired single cell modalities data, such as DVAE [18], scMVAE [19], DCCA [20], Seurat [21], MOFA+ [22] and scJoint [23] have limitted performance. Moreover, in most case, datasets are unpaired. Most modalities are sampled from the same sample or tissue. In this setting, the high efficiency computational methods are scarcely and most existing methods can be classified into two categories: one kind such as Conos [24], Seurat3 [25] and Liger [26] based nearest neighbor graph structure and matrix factorization. Specifically, methods that use mutual nearest neighbors(MNN) for data alignment become less scalable as datasets exceed one million cell. Additionally, these methods are mainly targeted towards integrating datasets of less complex tissues and may overcorrect fine-grained cell subpopulations in more complex tissues, resulting in the loss of power to reveal interesting biological variations. Other deep learning basesd methods (review in [27]) such as scJoint, sciCAN [28], Poral [29], scDART [30], Glue [31] outperform many methods in the situation of integrating unpaired scRNA-seq and scATAC-seq data. However, compared to the special-purpose networks tuned to the structure of problem space, the fully connected networks used by scJoint have weaker performance. Meanwhile, their performance varies owing to data noises, parameter settings and new input data. In order to reduce the domain discrepancy and improve the robustness of results, more powerful transfer learning methods and deep generative model should be designed.

**Fig 1:**
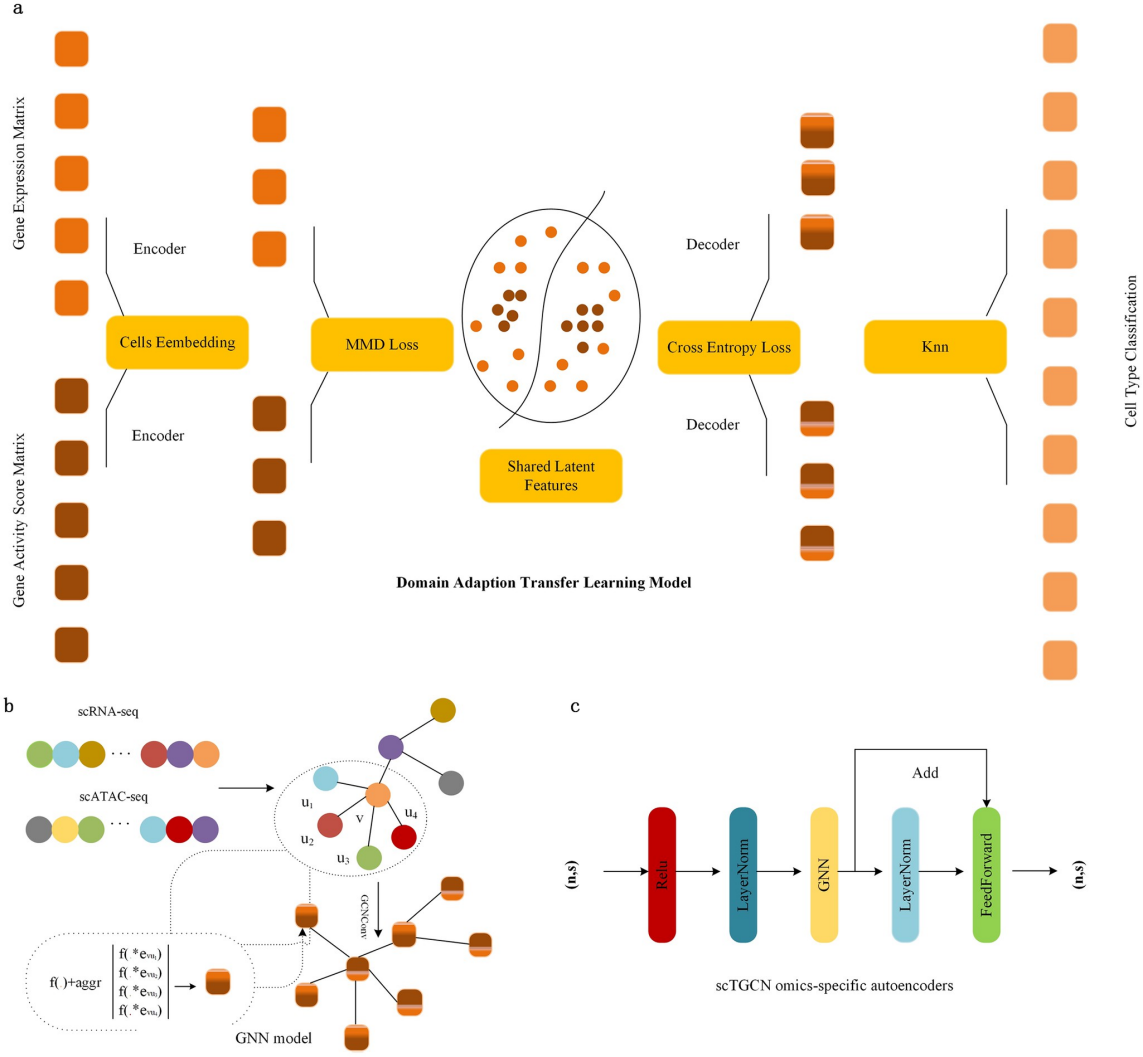
Workflow of scTGCN methods. scTGCN learns a hybrid graph of both scRNA-seq data and scATAC-seq cell mappings, in the hybrid graph, transfer learning is used to transfer cell type labels from scRNA-seq data to scATAC-seq data. a. Broad schematic of scTGCN workflow. The input of scTGCN consists of two modaities. One is scRNA-seq data and the other is scATAC-seq, scATAC-seq data is converted to gene activity scores calculated from the accessibility peak matrix. scTGCN is constructed upon the transfer learning, comprising three key modules: omics-specific autoencoder module, domain adaption transfer learning module and Graph convolutional network model. b. Graph convolutional network captures inter and intra modalities information by two stage of semisupervised learning. The graph convolutional layer aggregates information from neighboring nodes to update the features of each node. c. the input of scTGCN omics-specific autoencoders comprises n sub-vectors, characterized by dimensions of (n, s), the output also consists of n sub-vectors with dimensions of s.

In order to simultaneously address the above challenges. Here, we propose a single-cell transfer graph convolutional network model (Fig 1b), which regard the label transfer from scRNA-seq to scATAC-seq as the domain adaption problem in transfer learning (Fig 1a). This model formulates and aggregates cell-cell relationship and gene expression with graph convolutional network, which based on the kenetic relationships between the specific regulatory mechanisms of scRNAseq and scATAC-seq. And we explore the idea of MK-MMD-based adaption for learning transferable features in common embedding of scRNA-seq and scATAC-seq, enables our model to best leverage the domain-specific effects. scTGCN incorporates commmon information of two data modalities thorough a semisupervised paradigm to learn unlabeled scATAC-seq. Applying a wide range of single-cell omics datasets, we demonstrated that scTGCN have high label transfer accuracy. In addition, annotated data comfirm that integrative analysis by scTGCN can be applied for new cell type identification via tranfer learning.

## Materials and methods

### Methods overview

Here, we regard integrating multiple datasets as domain adaption problem in transfer learning. Using the GCN network to cotrain labeled data from the source domain (scRNA-seq) and unlabeled data from the target domain (scATAC-seq) following different distribution. By leveraging labeled scRNA-seq data, scATAC-seq data or other modalities, cell type can be classified accurately. And it is now publicly available as a Python software (https://github.com/kanyulongkkk/scTGCN), which is a flexibility and scalability tool for integrative analysis single cell multimodal data.

### Datasets

To evaluate the performance of the scTGCN, we applicated our methods on different tissues and organs from mouse and human multiomics data: The Tabula Muris atlas is a comprehensive resource for single-cell RNA sequencing (scRNA-seq) data [32]. It consists of data from 96,404 cells derived from 20 organs, using two different protocols: fluorescence-activated cell sorting (FACS), droplet. This atlas provides information on 73 distinct cell types. On the other hand, Cusanovich et al. presented an atlas for single-cell assay for transposase-accessible chromatin sequencing (scATAC-seq) data [33]. This atlas includes data from 81,173 cells obtained from 13 tissues. It identifies 29 different cell types, with one group annotated as ‘unknown’. Notably, there are 19 cell types that are common between the scRNA-seq atlas and the scATAC-seq atlas. Another dataset using multimodal measurements that simultaneously profiled gene expression levels or chromatin accessibility along with surface protein levels. These measurements were obtained through the CITE-seq [34] and ASAP-seq [35] techniques. Specificallly, we analyzed CITE-seq and ASAP-seq data commonly. In this experiment, cells were sequenced using both CITE-seq and ASAP-seq technologies in parallel.

### Data processing

For each input data, we denote labeled single-cell RNA sequencing (scRNA-seq) data as source domain data $X_{S}\in S^{m\times n_{s}}$, and the dataset that needs to be annotated as target domain data $X_{T}\in S^{m\times n_{t}}$, where *m* is the number of common gene features shared by *X*_*S*_ and *X*_*T*_, and *n*_*s*_ and *n*_*t*_ are the number of cells in the source and target domain data. The neural networks in scTGCN consists of one omic autoencoder layer, one graph convolution layer and two fully connected layers. After apply canonical correlation analysis [37] method between scRNA-seq and scATAC-seq data in both source and target domain dataset. We use the mutual nearest neighbor (MNN) method to construct the hybrid graph leveraging the topological characteristics of scRNA-seq and scATAC-seq data. The principle and formula of the MNN method [36] is designed to integrate and compare single-cell RNA sequencing datasets. Its core idea is to identify pairs of cells that are mutual nearest neighbors in two datasets for the purpose of data integration. The steps of the MNN method we used as follow: 1. Calculate the nearest neighbors of each cell in source dataset and target dateset, respectively. 2. Construct an integrated cell-cell association graph by comparing the nearest neighbor relationships. Formula derivation for constructing the graph: we have two datasets, with m cells in source dataset and n cells in target dataset. For cell i in source dataset and cell j in target dataset, the proximity between them is defined as:

$$\begin{array}{c} s_{ij}=\text{exp}\;(-\frac{{\left\|x_{i}-y_{j}\right\|}^{2}}{2\sigma^{2}}) \end{array}$$ (1)

$$\begin{array}{c} matrix_{ij}=\{\begin{array}{cc} 1, & \text{if}\;\text{cell}\;i\;\text{is}\;\text{a}\;\text{nearest}\;\text{neighbor}\;\text{of}\;\text{cell}\;j\;\text{and}\;\text{vice}\;\text{versa}\\ 0, & \text{otherwise} \end{array} \end{array}$$ (2)

Where (*x*_*i*_) and (*y*_*j*_) represent the expression feature vectors of cell i and cell j, respectively, and *σ* is a parameter controlling the proximity level. Based on the proximity, we can construct a proximity matrix S between source dataset and target dataset, where the element (*s*_*ij*_) indicates the proximity between cell i and cell j. Utilizing Canonical Correlation Analysis (CCA) to simultaneously project high-dimensional source and target data into a shared low-dimensional space. This enables the capture and representation of molecular patterns with similar biological significance across both datasets in a uniform manner. By identifying canonical correlation vectors between the two datasets. This process facilitates the identification of Most Nearest Neighbors (MNNs) by searching for pairs of cells that are closest to each other in the reduced-dimensional space after dimensionality reduction through CCA. The construction of an effective graph, represented as an adjacency matrix, leverages the topological characteristics between the source and target data for enhanced integration. One graph-based MNN is constructed between the source dataset and the target dataset, while another graph-based MNN is constructed solely on the source dataset. The hybrid graph is constructed by combining the two graphs, represented as $A^{M}\in R^{\left(n_{s}+n_{t}\right)\times \left(n_{s}+n_{t}\right)}$.

### Loss function

#### MMD loss

For scTGCN model, to best leverage the source domain data and target domain data, we construct an effective graph structure which is denoted by adjacent matrix. Then we use the multiple kernel variant of MMD (MK-MMD) proposed by Gretton et al [38], which is formalized to jointly maximize the two-sample test power and minimize the Type II error, i.e, the failure of rejection a false null hypothesis. The source domain dataset as well as the target domain dataset are regularization. Then a source-to-domain graph (inter-dataset) as well as an internal target graph (intra-datset) are constructed. The final hybrid graph is constructed by these two graph, which as the input for scTGCN model. *H*_*k*_ be the reproducing kernel Hilbert space with a characteristic kernel *k*. We project the scRNA-seq data and scATAC-seq data into common Hilbert space and co-train them. The MK-MMD is defined as

$$\begin{array}{c} D_{k}^{2}\left(p,q\right)≜{\left\|E_{x}\left[⌀\left(X_{S}\right)\right]-E_{x}\left[⌀\left(X_{T}\right)\right]\right\|}_{H_{k}}^{2} \end{array}$$ (3)

where *D*_*k*_(*p*, *q*) between probability distributions *p* and *q* is defined as the RKHS distance between the mean embeddings of *p* and *q*. The modality characteristic kernel associated with the gene feature map ⌀ is given by:

$$k\left(X^{S},X^{t}\right)=\left\langle ⌀\left(X^{S}\right),⌀\left(X^{t}\right)\right\rangle$$

This kernel is defined as the convex combination of *m* PSD kernels {*k*_*u*_}.

$$\begin{array}{c} \kappa ≜\{k=\sum_{u=1}^{m}\beta_{u}k_{u}:\sum_{u=1}^{m}\beta_{u}=1,\beta_{u}\geq 0,\forall u\} \end{array}$$ (4)

*β*_*u*_ is the parameter to limit the multi-kernel. *k* is characteristic. And *k* can leverage different kernel to best align scRNA-seq and scATAC-seq.

#### Cosine similarity loss

Each batch is represented as *C*, that is, $C={\left\{C^{s}\right\}}_{s=1}^{S}\cup {\left\{C^{t}\right\}}_{t=1}^{T}$, where *C*^*s*^ and *C*^*t*^ have C cells. After dimension reduction and feature alignment by MMD loss, cosine similarity loss is applied to the embedding layer outputs from *C*^*t*^. For each batch, cosine similarity attempts to maximize the similarity between RNA embedding and ATAC embedding. For a pair of general embedding vectors (*i*, *j*), the cosine similarity is defined as

$$\text{cos}\;\left(i,j\right)=\frac{\left\langle i,j\right\rangle }{\left\|u\|\|v\right\|},$$

where we choose the cosine similarity with the highest cosine scores. The loss is given by

$$\begin{array}{c} {\text{Loss}}_{\text{cosine}}=\frac{1}{C}\frac{C^{s}\cdot C^{t}}{\|C^{s}\left\|\right\|C^{t}\|} \end{array}$$ (5)

#### Cross entropy loss

For every *C*^*s*^ with cell type annotations $\left\{y_{t}^{s}\right\}$, we use cross entropy loss to predict scATAC-seq cell type from scRNA-seq data. The loss is given by

$$\begin{array}{c} {\text{Loss}}_{\text{cross}}=-y\;\text{log}\;\hat{y}-\left(1-y\right)\;\text{log}\;\left(1-\hat{y}\right) \end{array}$$ (6)

where *y* is the label of scRNA-seq data, and $\hat{y}$ is the probability distribution of cell type in scRNA-seq data.

### scTGCN method

#### Omics-specific autoencoder

Our architecture of an omics-specific autoencoder (Fig 1c) typically consists of an encoder and a decoder. The encoder compresses the input omics data into a lower-dimensional latent space representation, while the decoder reconstructs the original input from this latent representation. The key advantages of our omics-specific autoencoder is its ability to learn meaningful features from omics data. The latent space representation obtained from the encoder can reveal important biological patterns and relationships between scRNA-seq data and scATAC-seq data. Our omics-specific autoencoder architecture represented as

$$\begin{array}{c} Z=f_{\text{encoder}}\left(X\right) \end{array}$$ (7)

$$\begin{array}{c} \hat{X}=f_{\text{decoder}}\left(Z\right) \end{array}$$ (8)

*X*: Input data matrix representing single-cell omics data, $X\in R^{N\times D}$, where *N* is the number of cells.

*D*: The number of features.

*Z*: Low-dimensional representation in the latent space obtained through the encoder, $Z\in R^{N\times K}$, where *K* is the dimension of the latent space.

$\hat{X}$ : Reconstructed input data obtained through the decoder mapping back to the data space.

*f*_encoder_: Encoder function mapping the input data to the latent space.

*f*_decoder_: Decoder function mapping the latent representation back to the original data space.

#### Semi-supervised transfer learning model

We apply GCN [39] for semi-supervised transfer learning and transfer labels from source domain to target domain. Each cell is represented as a node. The scRNA-seq data have known cell types annotations. And the scATAC-seq have unknown cell type annotations. The objective of GCN is to predict the cell type annotation of scATAC-seq by using not only the features of each cell but also the information leveraging source and target data. Our GCN model has two inputs: one is the hybrid graph denoted by the adjacency matrix $A^{M}\in R^{N\times N}$, and the other is the feature matrix $X\in R^{m\times N}$, where *N* = *n*_*s*_ + *n*_*t*_ and *m* is the common gene features shared by scRNA-seq and scATAC-seq. The input matrix is represented as

$$\begin{array}{c} X=[\begin{array}{c} X_{S}\\ X_{T} \end{array}]\in R^{m\times (n_{s}+n_{t})} \end{array}$$ (9)

We define adjacent matrix *A*^*M*^ as:

$$\begin{array}{c} \tilde{A}={\overset{ˇ}{D}}^{-\frac{1}{2}}\hat{A}{\overset{ˇ}{D}}^{-\frac{1}{2}}={\overset{ˇ}{D}}^{-\frac{1}{2}}\left(A^{H}+I\right){\overset{ˇ}{D}}^{-\frac{1}{2}} \end{array}$$ (10)

Here, *I* is the identity matrix, $\hat{A}$ = *A*^*H*^ + *I*, *D* is the diagonal matrix of $\hat{A}$, and a multi-layer Graph Convolutional Network with the following layer-wise propagation rule:

$$\begin{array}{c} H^{\left(l+1\right)}=\sigma \left({\overset{ˇ}{D}}^{-\frac{1}{2}}\tilde{A}{\overset{ˇ}{D}}^{-\frac{1}{2}}H^{l}W^{l}\right) \end{array}$$ (11)

*W*^*l*^ is the weight matrix of the *l*-th layer. *σ*(.) denote an activation function. *H*^*l*^ is the matrix of activations in the *l*^*th*^ layer.

Each layer is defined as:

$$\begin{array}{c} H^{l+1}=f\left(H^{l},\tilde{A}\right)=\sigma \left(\tilde{A}H^{l}W^{l}\right) \end{array}$$ (12)

The network take an input *H*^0^ and passes it through multiple layers, each with its own weight matrix *W*^*l*^ and non-linear activation function *σ*(). The goal is to predict the labels for cells in target domain. These labels are represented as class indicators. The forward propagation is denoted as:

$$\begin{array}{c} \hat{Y}=f\left(H^{l},\tilde{A}\right)=softmax\left(\left(\tilde{A}ReLU\left(\tilde{A}X^{T}W^{0}\right)W^{l}\right)\right) \end{array}$$ (13)

The softmax activation function is denoted as:

$$\begin{array}{c} softmax\left(.\right)=\frac{exp(.)}{\sum exp(.)} \end{array}$$ (14)

In a neural network with input-to-hidden and hidden-to-output layers, the input-to-hidden weight matrix *W*^0^ project the input data onto the *h* dimensional hidden layer. The rectified linear unit (ReLU) activation function is applied to the output of the input-to-hidden layer, which is then fed into the hidden-to-output weight matrix *W*^*l*^ to produce the predicted probabilities of cell labels $\hat{Y}$. After training the model by GCN, the co-embeding space of scRNA-seq and scATAC-seq modalities which similarity to each other closely enough. Then we calculate the Euclidean distance of the embedding vectors. We choose 30 nearest neighbors to predict the label transfer accuracy of scATAC-seq data.

### Evaluation indicators

#### Silhouette coefcients

$$\begin{array}{c} s_{\text{modality}}=\frac{b(i)-a(i)}{\text{max}\;(a(i),b(i))} \end{array}$$ (15)

*s*_modality_: it measures how similar an object is to its own cluster compared to other clusters.

*b*(*i*): The average distance from the sample i to the samples in the nearest different cluster.

*a*(*i*): The average distance from the sample i to all other samples in the same cluster.

max(*a*(*i*), *b*(*i*)): The maximum value between *a*(*i*) and *b*(*i*) used to normalize the difference between b(i) and a(i).

$$\begin{array}{c} s_{\text{cellTypes}}=\frac{b(i)-a(i)}{\text{max}\;(a(i),b(i))} \end{array}$$ (16)

*s*_cellTypes_: it measures how similar an object is to its own cluster compared to other clusters.

*b*(*i*): The average distance from the sample i to the samples in the nearest different cell type cluster.

*a*(*i*): The average distance from the sample i to all other samples in its own cell type cluster.

max(*a*(*i*), *b*(*i*)): The maximum value between *a*(*i*) and *b*(*i*) used for normalization.

$$\begin{array}{c} f1_{\text{score}}=\frac{2\cdot \left(1-s^{\prime }\text{modality}\right)\cdot s^{\prime }\text{cellTypes}}{1-s^{\prime }\text{modality}+s^{\prime }\text{cellTypes}} \end{array}$$ (17)

*f*1_score_: *f*1 score for silhouette coefficients; a measure that combines the two silhouette coefficients to evaluate clustering performance.

*s*′modality: *f*1 score for silhouette coefficients; a measure that combines the two silhouette coefficients to evaluate clustering performance.

*s*′cellTypes: Normalized cell-type silhouette coefficient.

A higher F1 score indicates that the joint embeddings from different methods have better alignment of modalities and better perservation of biological signals, and thus are more informative for downstream analysis.

#### Average silhouette width

$$\begin{array}{c} \text{cell}\;\text{type}\;\text{ASW}=\frac{1}{2}(\frac{\sum_{i=1}^{N}s\left(i\right)}{N+1}) \end{array}$$ (18)

cell type ASW: Represents the Average Score Weighting for a specific cell type, used to evaluate the characteristics or performance of that cell type.

*N*: Denotes the number of samples, which refers to the number of cell instances included in the calculation.

s(i): Represents the score or feature value for the *i*-th cell sample, such as gene expression levels, activity.

$\sum_{i=1}^{N}s\left(i\right)$ : This summation calculates the total score of all cell samples from the 1st to the *N*-th sample.

*N* + 1: This is the denominator in the average calculation, which adds 1 to the sample count to potentially avoid division by zero or for normalization purposes.

$$\begin{array}{c} \text{omics}\;\text{layer}\;\text{ASW}=\frac{1}{M}\sum_{j=1}^{M}\text{omics}\;\text{layer}\;{\text{ASW}}_{j} \end{array}$$ (19)

omics layer ASW: Represents the average silhouette width across all omics layers, indicating the quality of mixing among different cell types.

Cell type ASW [42] and Omics layer ASW [42] have a range of 0 to 1, and higher values indicate better cell type resolution and better omics mixing.

#### Neighbor consistency

$$\begin{array}{c} NC=\frac{1}{N}\sum_{i=1}^{N}|\frac{NNS(i)\cap NNI(i)}{NNS(i)\cup NNI(i)}| \end{array}$$ (20)

*NC*: Stands for “Normalized Coefficient,” typically used to measure the similarity between two sets.

*N*: Represents the total number of samples or data points. It is used as the denominator for calculating the average.

*i*: An index representing the current sample number being calculated, iterating from 1 to (N).

*NNS*(*i*): Denotes the Nearest Neighbors Set for the (*i*^*th*^) sample, which usually refers to the set of other samples that are close to the (*i*^*th*^) sample.

*NN*(*i*): Represents the “Relevant Items Set” for the (*i*^*th*^) sample, typically indicating the set of other samples that are relevant to the (*i*^*th*^) sample.

∩: Indicates the intersection of two sets. (*NNS*(*i*) ∩ *NNI*(*i*)) refers to the elements that are present in both sets.

∪: Indicates the union of two sets. (*NNS*(*i*) ∪ *NNI*(*i*)) refers to the elements that are present in at least one of the sets.

Neighbor consistency [43] was used to evaluate the preservation of single-omics data variation.

#### FOSCTTM

$$\begin{array}{c} FOSCTTM=\frac{1}{2N}(\sum_{i=1}^{N}n\left(i\right)1\cdot \frac{1}{N}+\sum {i=1}^{N}n{\left(i\right)}_{2}\cdot \frac{1}{N}) \end{array}$$ (21)

$$\begin{array}{c} n{\left(i\right)}_{1}=|j|d\left(x_{j},y_{i}\right)<d\left(x_{i},y_{i}\right)| \end{array}$$ (22)

$$\begin{array}{c} n{\left(i\right)}_{2}=|j|d\left(x_{i},y_{j}\right)<d\left(x_{i},y_{i}\right)| \end{array}$$ (23)

*FOSCTTM*: Represents the Fuzzy Overlap Score for Cross-Omics Ties and Mappings, which quantifies the extent of overlap or mixing among different omics layers.

*N*: The total number of samples or observations in the dataset.

*n*(*i*)_1_: The count of samples (*j*) for the (*i*^*th*^) sample where the distance (*d*(*x*_*j*_, *y*_*i*_)) is less than the distance (*d*(*x*_*i*_, *y*_*i*_)). This measures the number of close neighbors within a specified distance from the (*i*^*th*^) sample.

*n*(*i*)_2_: The count of samples (*j*) for the (*i*^*th*^) sample where the distance (*d*(*x*_*i*_, *y*_*j*_)) is less than the distance (*d*(*x*_*i*_, *y*_*i*_)). This similarly measures the number of close neighbors within a specified distance from the (*j*^*th*^) sample.

*d*(*x*, *y*): Represents the distance function between two points (*x*) and (*y*). The specific context of the distance metric.

FOSCTTM [44] was used to evaluate the single-cell level alignment accuracy.

#### Seurat alignment score

$$\begin{array}{c} SAS=1-\frac{\bar{x}-K_{N}}{K-K_{N}} \end{array}$$ (24)

*SAS*: represents the Seurat alignment score.

$\bar{x}$ : is the mean value being evaluated.

*K*: is the total number of observations.

*K*_*N*_: is the total number of observations.

Seurat alignment score [45] was used to evaluate the extent of mixing among omics layers.

#### Label transfer accuracy

To evaluate the accuracy of label transfer between scRNA-seq and scATAC-seq data, label transfer accuracy measure the percentage of cells that were correctly labeled after label transfer. It was computed for the common cell types between the two modalities. We assessed the accuracy of label transfer using two measures: (1) overall accuracy rate and (2) cell-type classification F1 score. The overall accuracy rate was computed by considering only the shared cell types between scRNA-seq and scATAC-seq data. The cell-type classification F1 score represents the harmonic mean of precision and recall for each cell type.

## Results

scTGCN cotrain labeled scRNA-seq and unlabeled scATAC-seq data by transfer learning. We compared scTGCN with Seurat, Conos, GLUE and scJoint for label transfer accuracy. We demonstrate the performance of methods by integrating two mouse cell atlases: Tabula Muris atlas for scRNA-seq data and the atlas in Cusanovich et al. [46] for scATAC-seq data. One Multimodal data (CITE-seq and ASAP-seq PBMC data). We perform comprehensive ablation studies on the whole dataset, and the results show the effectiveness of (S1–S5 Tables) different components.

### Integration of scRNA-seq and scATAC-seq on subset of atlas data

**Fig 2:**
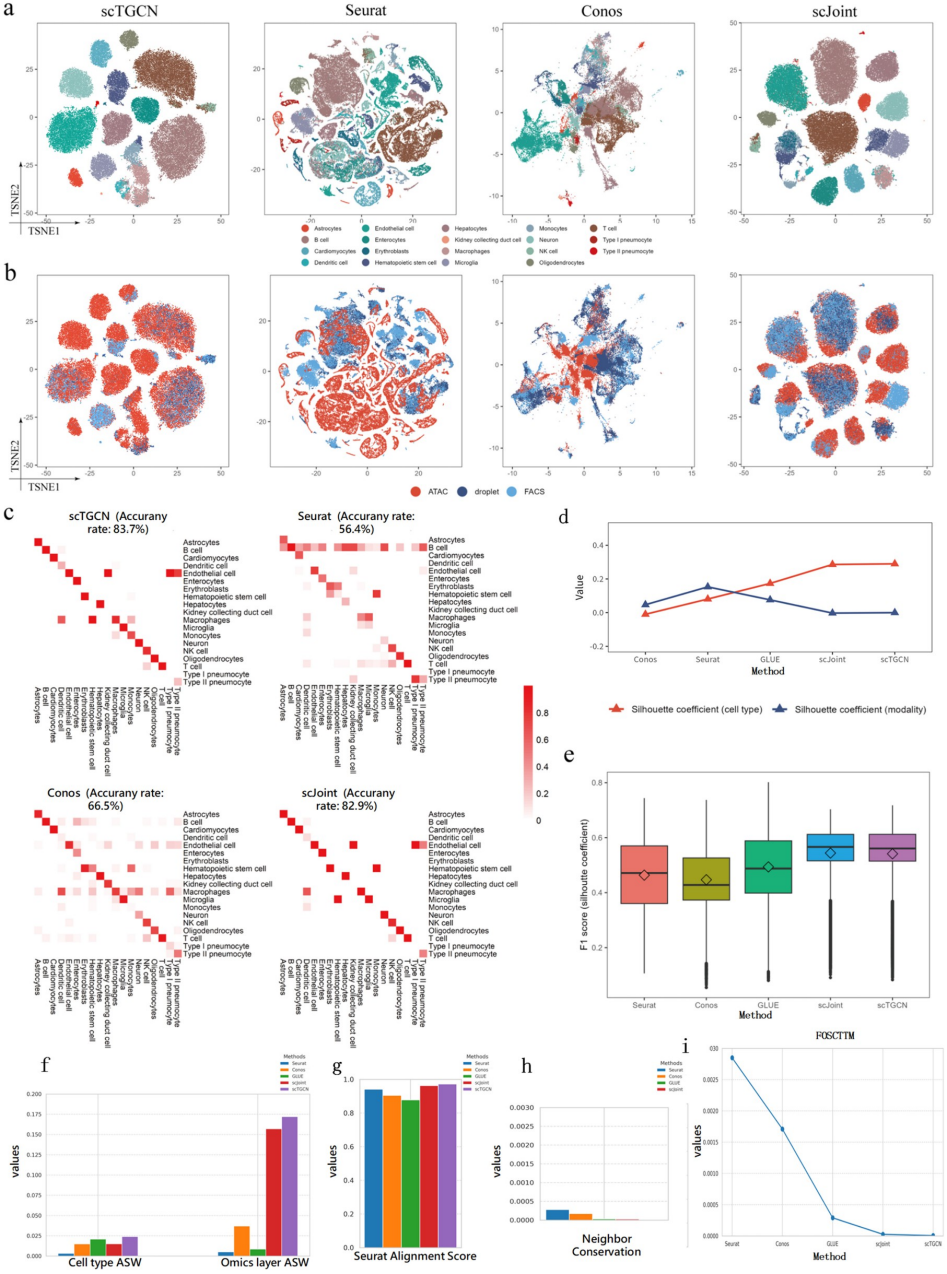
Integration and analysis of overlapping cell types from scRNA-seq and scATAC-seq modalities in mouse cell atlas subset data. a, t-SNE Visualization of scTGCN, Seurat, Conos and scJoint with cell types defined in Cusanovich et al. [46] as Color Labels. b, t-SNE Visualization of scTGCN, Seurat, Conos and scJoint with three protocols. c, Predicted cell types and fractions of agreement with Cusanovich et al. [46] for scTGCN, Seurat, Conos and scJoint. A clearer diagonal structure indicates a higher level of agreement. d, Comparison of modality silhouette coeffcient and cell-type silhouette coeffcient of different methods. e, Comparison of F1 scores of different methods. f, Comparison of ASW of different methods. g, Comparison of SAS of different methods. h, Comparison of NC of different methods. i, Comparison of foscttm of different methods.

The full of the atlas data contain 73 (96,404 cells from 20 organs, two protocols) and 29 (81,173 cells from 13 tissues) cell types, of which 19 are common between the two modalities. Our initial evaluation focused on this 19 overlap common cell of the atlas data, which contains 101,692 cells from the 19 overlapping cell types. To evaluate the accuracy of our method, we transfered cell-type labels from scRNA-seq to scATAC-seq and compared the results with the original labels from Cusanovich et al. [46]. The joint visualizations provided a better grouping of the cells in terms of previously defined cell types produced by our method (Fig 2a) and effectively mixed the three protocols (ATAC, droplet, FACS) than other methods (Fig 2b). In terms of label transfer accuracy, scTGCN accurately assigns 83.7% of the cells to their correct cell types, which is 27.3% (T-statistic: 88.50800736894429, p-value: 2.9632047415877106e-13), 17.2% (T-statistic: 28.352286180806594, p-value: 2.5883875914327767e-09), 0.8% (T-statistic: 3.130495168499689, p-value: 0.01400480331859369) and 8.5% (T-statistic: 15.761570503055584, p-value: 2.623343447224337e-07) higher compared to Seurat, Conos, scJoint and GLUE (Fig 2c and S1 Fig) respectively. These performance are supported by the quantitative evaluation metrics. Specifically, scTGCN exhibits significantly higher cell-type silhouette coefficients compared to all other methods, while demostrating similar modality silhouette coefficients as scJoint and Conos (Fig 2d). Moreover, scTGCN achieves the highest median F1 score of silhouette coefficients nearly the same as scJoint (Fig 2e), striking a better balance between mitigating technological variations across modalities and preserving the cell-type signals. In comparision of cell type ASW and omics layer ASW, scTGCN also have the best peformance (Fig 2f), indicating better cell type resolution and better omics mixing. Although scTGCN performs slightly lower than other methods in neighbor conservation (Fig 2h), our method have the lowest foscttm (Fig 2i) values and the best SAS (Fig 2g) compared with all state of art methods, showing high consistency of feature embeddings in multi omics integration.

### Integration of scRNA-seq and scATAC-seq on full atlas data

**Fig 3:**
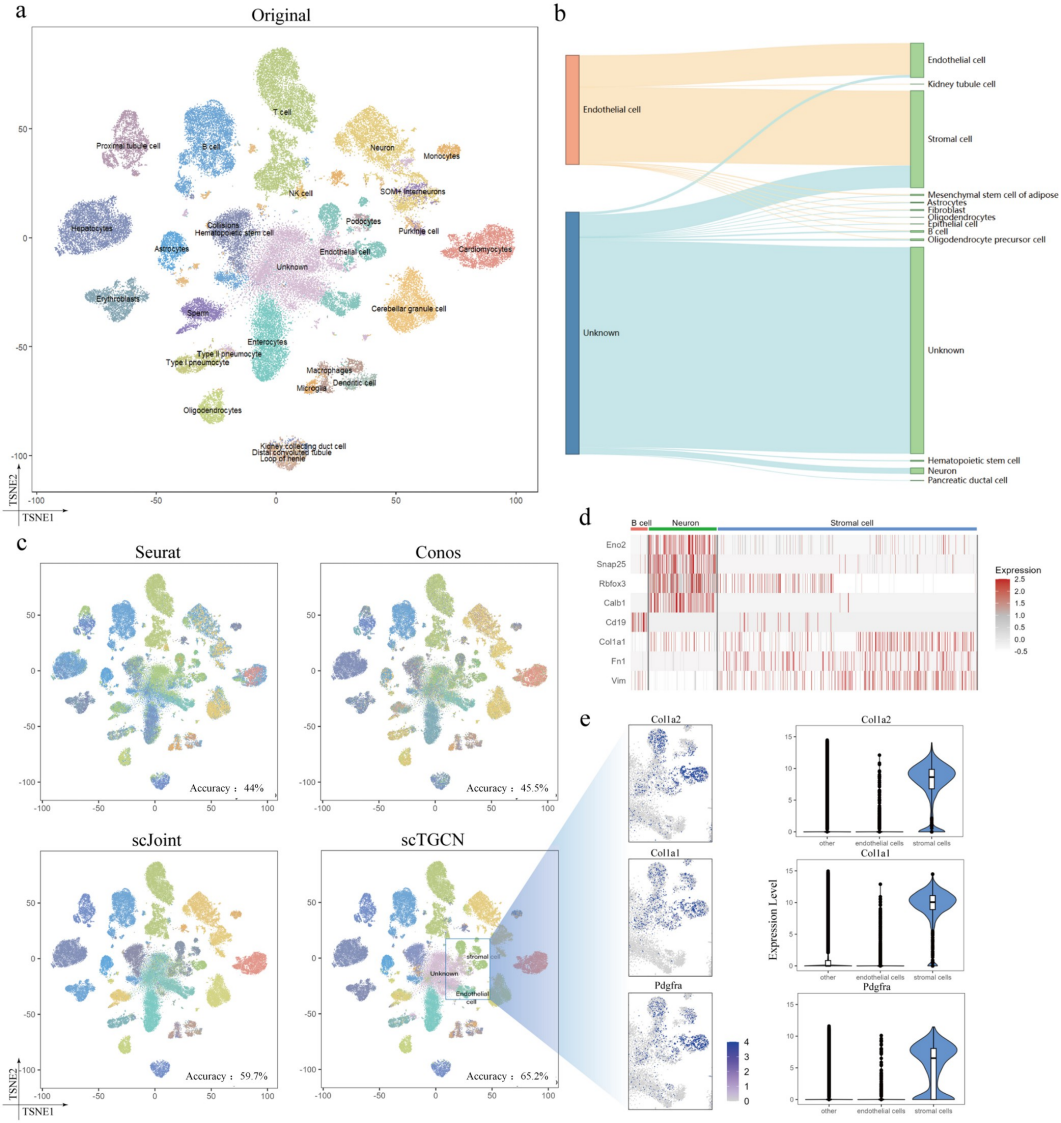
Analysis of large scale full mouse cell atlas data. a, t-SNE plots generated from the top 100 dimensions resulting from singular value decomposition of TF-IDF transformed ATAC-seq data, with data points colored according to their original labels. b, Refining scATAC-seq annotations in heterogeneous atlas data. c, The transferred labels accuracy of each cell type identified by different methods based on large-scale full atlas data. d, Gene expression levels of cd19 in B cells, Eno2, Snap25, Rbfox3, Calb1 in neuron cells, and Col1a1, Fn1, Vim in stromal cells. e, Marker expressions in stromal cells: Col1a2, Col1a1, and pdgfra. The left column displays the high-level gene activity scores and the right column exhibits the gene expression levels in endothelial, stromal cells, and others from scRNA-seq.

**Fig 4:**
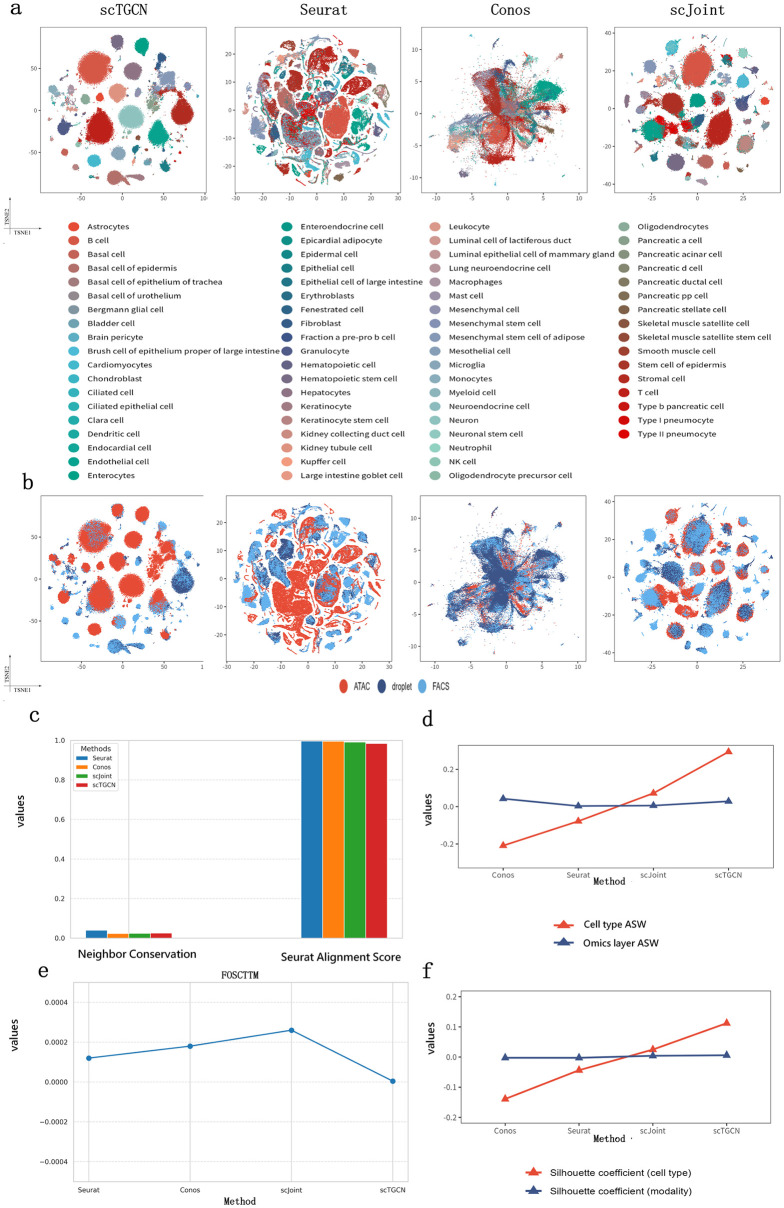
Integration of full mouse atlas data. a, t-SNE Visualization of scTGCN, Seurat, Conos and scJoint with cell types defined in Cusanovich et al. [46] as Color Labels. b, t-SNE Visualization of scTGCN, Seurat, Conos and scJoint with three protocols. c, Comparison of NC and SAS of different methods. d, Comparison of cell type ASW and modality ASW of different methods. e, Comparision of foscttm of different methods. f, Comparison of modality silhouette coeffcient and cell-type silhouette coeffcient of different methods.

We then tackled the more complex task of integrating large-scale full atlas data using mouse atlases. Given that the scRNA-seq atlas encompasses a larger number of cell types compared to the scATAC-seq atlas, we employed this application to showcase how transferred labels can enhance and provide novel annotations to ATAC cells. To compare the results with the original labels, we constructed t-SNE plots following the methodology described by Cusanovich et al. [46]. Specifically, We observe that scTGCN enables us to assign cell labels to those originally categorized as ‘unknown’ in Cusanovich et al. [46], with a probability score exceeding 0.60. These cells are distinctly grouped in the tSNE visualization of scTGCN’s embedding space (Fig 3c), primarily falling into clusters of endothelial cells and stromal cells. we utilized singular value decomposition on the term frequency-inverse document frequency (TF-IDF) transformation of the scATAC-seq peak matrix (Fig 3a). Remarkably, we observed that scTGCN consistently assigned labels to cells in close proximity within the ATAC visualization space, exhibiting a higher degree of consistency compared to alternative methods. Upon further examination of the transferred labels, we observed that scTGCN identified a new cluster of cells (initially labeled as ‘unknown’ or ‘endothelials’) as ‘stromal cells’ (4,014 cells) (Fig 3b). In (Fig 3d), we collect enriched genes which discovery and justify by Schaum et al. cd19 in B cell, Eno2, Snap25, Rbfox3, Calb1 in Neuron cell and Col1a1, Fn1, Vim in Stromal cell. Using cell-type markers identified from the scRNA-seq data, the aggregated gene activity scores of these ATAC cells exhibit clear differential expression patterns across different cell types. Additionaly, scTGCN assigned one cell types, namely ‘stromal cells’, which were not originally identified in the ATAC labels. These cells exhibited elevated gene activity scores for Col1a2, Col1a1, Pdgfra, all of which are genes with high expression levels in stromal cells, but low expression levels in endothelial cells, as indicated by the scRNA-seq data (Fig 3e). Consequently, the newly assigned annotations demonstrate a higher degree of consistency with the expression patterns of these genes. Meanwhile, the full mouse atlas data containing nearly millions of cells. The sheer data volume, extensive cellular diversity, low coverage per cell, imbalanced cell type distributions, and the fact that achieving this integration more challenge. Using an efficient multistage transfer learning strategy for scTGCN Methods, meanwhile we successfully integrated the gene expression and chromatin accessibility data into a unified multi-omics mouse cell atlas (Fig 4a and 4b). By employing a neural network architecture optimized through graph convolutional aggregation, scTGCN offers excellent scalability at a sublinear time complexity, demonstrating its potential for mouse full atlas at the atlas scale (S3 Fig). Moreover, scTGCN achieves the highest cell type ASW, omics layer ASW (Fig 4d). The cell type silhouette coefficients score and modality silhouette coefficients score (Fig 4f) also perform well, striking a better balance between mitigating technological variations across modalities and preserving the cell-type signals. Although scTGCN performs slightly lower than other methods in neighbor conservation (Fig 4c) and seurat alignment score, our method have the lowest foscttm (Fig 4e) values compared with all state of art methods, showing high consistency of feature embeddings in large full atlas multi omics integration.

### Integration of multimodal PBMC data

**Fig 5:**
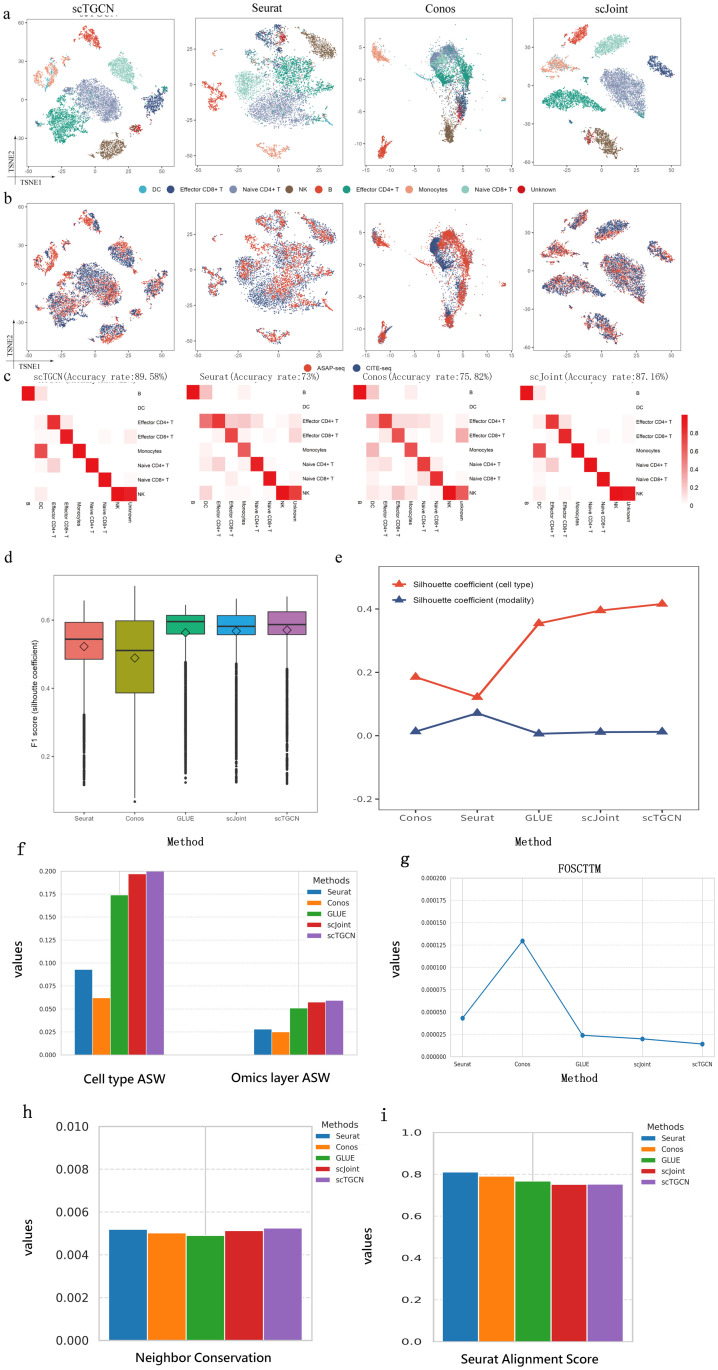
Integration of multimodal PBMC data. a, t-SNE visualizations of PBMC data, comparing scTGCN, Seurat, Conos and scJoint results. b, The plot represents different technology of scTGCN, Seurat, Conos and scJoint. c, Predicted cell types and fractions of agreement with true label. For scTGCN, Seurat, Conos and scJoint. A clearer diagonal structure indicates a higher level of agreement. d, Comparison of F1 scores of scTGCN, Seurat, Conos and scJoint. e, Comparison of modality silhouette coefcient and cell-type silhouette coefcient of different methods. f, Comparision of ASW of different methods. g, Comparision of foscttm of differet methods. h, Comparision of NC of differet methods. i, Comparision of SAS of different methods.

We demonstrate the versatility of scTGCN in integrating additional modality information from RNA-seq and ATAC-seq, making it applicable to experiments involving diverse biological conditions. Specifically, we explore the incorporation of multimodal measurements that simultaneously profile gene expression levels and chromatin accessibility with surface protein levels. These measurements can be obtained using techniques such as CITE-seq (Stoeckius et al) and ASAP-seq (Mimito et al), Upon examining the tSNE plots, we observe that our method successfully achieves improved clustering of cells based on pre-defined cell types compared to other methods (Fig 5a), integrates the two protocols (ASAP-seq and CITE-seq) (Fig 5b and S2 Fig), meanwhile our approach effectively combines the different modalities, resulting in enhanced grouping of cells in accordance with their known cell types (Fig 5c). In terms of label transfer accuracy, scTGCN accurately assigns 83.7% of the cells to their correct cell types, which is 16.58% (T-statistic: 37.79914638950812, p-value: 2.634098238076824e-10), 13.76% (T-statistic: 11.519586324068081, p-value: 2.9251972993356135e-06), 2.42% (T-statistic: 5.357970057517547, p-value: 0.0006793239768526269) and 16.82% (T-statistic: 39.000441133430144, p-value: 2.053335507327095e-10) higher compared to Seurat, Conos, scJoint and GLUE (Fig 5c and S2 Fig) respectively. From a quantitative perspective, these findings are reinforced by scTGCN’s superior cell-type silhouette coefficients (Fig 5e) and modality silhouette (Fig 5e) and F1 score (Fig 5d). In cell type ASW and modality ASW (Fig 5f), neighbor conservation (Fig 5h) and seurat alignment score (Fig 5i), our method performs well among all methods. Meanwhile scTGCN have the lowest foscttm (Fig 5g) values compared with all state of art methods, showing high consistency of feature embeddings in PBMC multi omics integration.

## Discussion

The scTGCN framework is a novel end-to-end and hypotheses-free framework for integrating unpaired single-cell multi-omics data with high accuracy and robustness. Our method is achieved by combining omics-specific autoencoders (Fig 1c) with graph convolutional networks and transfer learning, which allows for the integration of different types of omics data that have different distribution. One unique feature of scTGCN is that it models regulatory interactions (S5 and S6 Figs) explicity across different omics layers. It means that scTGCN can infer regulatory relationships between different types of omics data, even when they are unpaired.

Single-cell omics technologies have revolutionized our ability to study the individual components of complex biological systems at a cellular level [40, 41]. However, with the exponential growth of sigle-cell data, there is an urgent need to leverage existing well-characterized datasets as source to ensure relaiable and consistent annotations of target data. To address this challenge, our model approaches the integration of scRNA-seq and scATAC-seq as a domain adaption problem in transfer learning. Meanwhile, we explore multiple kernels for adapting deep representations, which enhances adaption effectiveness between the mean embeddings of scRNA-seq and scATAC-seq. We provide evidence that scTGCN can accurately transfer cell type annotations across dataset from different tissues, species and molecular layers, such as scRNA-seq and scATAC-seq. we also provide evidence that scTGCN facilitates the identification of new cell subtypes (S4 Fig), and can help researchers reannotate cell types in scATAC-seq data more accuracy.

As single-cell atlasing data advances and generates, the scalability of computational methods become increasingly important [46, 47]. Many sophisticated methods, such as the recently developed scIAE [48], GLOBE [49], DURIAN [50] and CLEAR [51] provide many flexible frameworks for joint modeling of variation across both modalities, technologies and conditions. We assessed the scalability of scTGCN methods on large single-cell datasets generate by Cusanovich et al [46] and Schaum et al [47]. compared to Seurat, Conos, scJoint and GLUE, the label transfer results demonstrate that scTGCN is an effective and scalable tool for analyzing large single-cell datasets, meanwhile providing valuable insights into complex biological system at the single-cell level. Despite scTGCN methods current focus on integration scRNA-seq and scATAC-seq, the generative distributions can also be adapted to include other types of omics layers, such as protein abundance [52], histone modification [53] and methylation data [54].

scTGCN is a method that has several technical advantages over other methods for integration analysis of single-cell data. Firstly, scTGCN explore the idea of MK-MMD-based adaption for learning transferable features in graph convolutional network, which co-train labeled scRNA-seq and unlabeled scATAC-seq into a semisupervised paradigm efficiently. Secondly, scTGCN nonlinearly propagates feature information from neighboring cells in the hybrid graph at every layer, which enables it learn the topological cell relationships and consider higher-order relations between cells. This helps to improve the accuracy of label transfer and enables scTGCN to capture more complex biological patterns.

Despite the success application for unpaired scRNA-seq and scATAC-seq integration. The scTGCN currently only focus on cell classification and identification. However, single cell data also be used for other tasks such as trajectory inference, cell-cell interaction analysis and time-series analysis. Meanwhile, the deep learning model are considered as black boxes [55–58] because it can be difficult to understand how they arrive at their predictions or decisions.

In brief, scTGCN is a novel transfer learning approach based graph convolutional network that enables the integration and analysis of larger-scale atlas-level single-cell multiomics data. The method is versatile and effectively combine different types of measurements, even if the data is unpaired. Compared to other methods, scTGCN has been shown to achieve higher accuracy in label transfer and can generate visualizations that remove technical noise while retainging the biologically relevant information. By capturing diverse aspects of cell characteristics, scTGCN provides a more holistic view of cell funcitons and communication.

## Supporting information

S1 Data Data for the plots presented in Fig 2. Sheets A, B, C, and D correspond to Fig 2a and 2b. Sheet E to Fig 2c, Sheet F to Fig 2d, Sheet G to Fig 2e, Sheet H to Fig 2f, Sheet I to Fig 2g, Sheet J to Fig 2h, Sheet K to Fig 2i.
(XLSX)

S2 Data Data for the plots presented in Fig 3. Sheets A, B, and C correspond to Fig 3a. Sheet D to Fig 3b, Fig 3d and 3e. Sheets E, F, G, and H correspond to Fig 3c.
(XLSX)

S3 Data Data for the plots presented in Fig 4. Sheets A, B, C, and D correspond to Fig 4a and 4b. Sheet E to Fig 4d, Sheet F to Fig 4c, Sheet G to Fig 4c, Sheet H to Fig 4e, Sheet I to Fig 4f.
(XLSX)

S4 Data Data for the plots presented in Fig 5. Sheets A, B, C, and D correspond to Fig 5a and 5b. Sheet E to Fig 5c, Sheet F to Fig 5d, Sheet G to Fig 5e, Sheet H to Fig 5f, Sheet I to Fig 5g, Sheet J to Fig 5h, Sheet K Fig 5i.
(XLSX)

S5 Data Data for the plots presented in S1–S6 Figs. Sheet A to S1 Fig. Sheet B to S2 Fig. Sheet C to S3 Fig. Sheet D to S4 Fig. Sheet E to S5 and S6 Figs.
(XLSX)

S1 Table First-order sensitivity of each hyperparmeter. (PDF)

S2 Table Second-order sensitivity of each hyperparmeter. (PDF)

S3 Table Performance results of view ablation study on PBMC data. (PDF)

S4 Table Performance results of view ablation study on mouse subset data. (PDF)

S5 Table Performance results of view ablation study on mouse atlas data. (PDF)

S1 Fig a, tSNE visualization of the overlapping subset data from mouse cell atlases for GLUE, colored by cell type. b, tSNE visualization of the overlapping subset data from mouse cell atlases for GLUE, colored by technology. c, Label transfer accuracy in overlapping subset data from mouse cell atlases.
(TIF)

S2 Fig a, tSNE visualization of the PBMC data from mouse cell atlases for GLUE, colored by cell type. b, tSNE visualization of the overlapping subset data from PBMC data for GLUE, colored by technology. c, Label transfer accuracy in PBMC data.
(TIF)

S3 Fig The running time and memory consumption of different methods on mouse atlas subsets of 5,000–100,000 cells. (TIF)

S4 Fig The confidence score predicted by scTGCN on different types of 81,173 scATAC-seq cells. (PNG)

S5 Fig Gene regulatory network in mouse atlas data. Some gene-peak relationship data is sampled from our gene2peak file. Each sampled line of data is split into seven values, including chromosome information, start and end positions for genes and peaks, and correlation values. Nodes representing genes and peaks are added to the graph G, and edges are created between genes and peaks with weights set to the correlation values.
(TIF)

S6 Fig Gene peak relation score in mouse atlas data. A line is drawn connecting the start and end positions of the gene and peak. A circular marker is placed at the peak position with a label showing the correlation score. The gene name is displayed near the circular marker for better identification.
(TIF)
