# Supplementary material for: Integration of unpaired single cell omics data by deep transfer graph convolutional network
Source: PLoS Comput Biol. 2025 Jan 16;21(1):e1012625. doi: 10.1371/journal.pcbi.1012625 (PMC11778791; doi:10.1371/journal.pcbi.1012625)
Supplement: S1 Table — (PDF) [file pcbi.1012625.s006.pdf]

S1 Tables. First-order sensitivity of each hyperparmeter

	S1	S1_conf
lr_stage	0.5972	1.1658
lr_decay_epoch	0.2203	0.5483
epochs_stage	0.0102	0.2213
p	-0.4523	1.6112
momentum	0.0132	0.0037
center_weight	-0.0130	0.33320
