# Supplementary material for: Integration of unpaired single cell omics data by deep transfer graph convolutional network
Source: PLoS Comput Biol. 2025 Jan 16;21(1):e1012625. doi: 10.1371/journal.pcbi.1012625 (PMC11778791; doi:10.1371/journal.pcbi.1012625)
Supplement: S2 Table — (PDF) [file pcbi.1012625.s007.pdf]

S2 Tables. Second-order sensitivity of each hyperparmeter

	S2	S2_conf
(lr_stage, lr_decay_epoch)	-1.1222	0.5222
(lr_stage, epochs_stage)	-1.1517	0.4067
(lr_stage, p)	-1.2151	0.4614
(lr_stage, momentum)	-1.8718	3.0056
(lr_stage, center_weight)	0.0834	2.0006
(lr_decay_epoch, epochs_stage)	0.0731	1.9667
(lr_decay_epoch, p)	1.7101	1.9590
(lr_decay_epoch, momentum)	-0.3682	0.3593
(lr_decay_epoch, center_weight)	0.6809	1.0657
(epochs_stage, p)	-0.3833	0.3429
(epochs_stage, momentum)	-0.3634	0.1112
(epochs_stage, center_weight)	-0.3223	0.3348
(p, momentum)	0.0630	2.6654
(p, center_weight)	1.7002	1.999
(momentum, center_weight)	1.1101	2.6641
