# Supplementary material for: Integration of unpaired single cell omics data by deep transfer graph convolutional network
Source: PLoS Comput Biol. 2025 Jan 16;21(1):e1012625. doi: 10.1371/journal.pcbi.1012625 (PMC11778791; doi:10.1371/journal.pcbi.1012625)
Supplement: S3 Table — (PDF) [file pcbi.1012625.s008.pdf]

S3 Tables. Performance results of view ablation study on PBMC Data.

	Abandon View		NMI	ARI	Silhouette	Purity	Davies-Bouldin Index	Jaccard Index	Integration ACC
PBMC Dataset	Lr_stage		Default Lr_decay_epoch = 10						
	Basic_Loss	0.01	0.5914	0.1209	0.01395	1.0516	2.91023	0.0	0.923
		0.001	0.5478	0.1120	0.00633	1.2310	3.0661	0.0003	0.912
	Basic_MMD_Loss	0.01	0.5916	0.1217	0.01521	1.0840	2.71021	0.00010	0.931
		0.001	0.5480	0.1184	0.00634	1.2310	2.9641	0.0090	0.914
PBMC Dataset	Lr_decay_epoch		Default Lr_stage = 0.01						
	Basic_Loss	1	0.5221	0.101	0.00891	0.944	3.1637	0.0	0.826
		10	0.5914	0.1209	0.01395	1.0516	2.9102	0.0	0.923
		20	0.5369	0.1072	0.00326	1.0504	3.0966	0.00002	0.850
		30	0.5005	0.0711	0.00310	1.0064	3.9656	0.0	0.606
	Basic_MMD_Loss	1	05229	0.092	0.00904	1.003	3.1610	0.0	0.834
		10	0.5916	0.1217	0.01521	1.0840	2.7102	0.00010	0.931
		20	0.5596	0.1143	0.00985	1.0648	3.0209	0.00003	0.907
		30	0.5449	0.1013	0.00834	1.0600	3.5903	0.00001	0.825
