# Supplementary material for: Integration of unpaired single cell omics data by deep transfer graph convolutional network
Source: PLoS Comput Biol. 2025 Jan 16;21(1):e1012625. doi: 10.1371/journal.pcbi.1012625 (PMC11778791; doi:10.1371/journal.pcbi.1012625)
Supplement: S4 Table — (PDF) [file pcbi.1012625.s009.pdf]

**S4 Tables. Performance results of view ablation study on Mouse Subset Data.**

| Dataset | Abandon View | | NMI | ARI | Silhouette | Purity | Davies-Bouldin Index | Jaccard Index | Integration ACC |
|---|---|---|---|---|---|---|---|---|---|
| Mouse Subset Dataset | Lr_stage | | Default Lr_decay_epoch = 10 | | | | | | |
| | Basic_Loss | 0.01 | 0.672 | 0.186 | 0.072 | 1.111 | 2.270 | 0.0155 | 0.829 |
| | | 0.001 | 0.657 | 0.172 | 0.030 | 1.111 | 2.991 | 0.0103 | 0.817 |
| | Basic_MMD_Loss | 0.01 | 0.700 | 0.201 | 0.092 | 1.019 | 2.231 | 0.0131 | 0.834 |
| | | 0.001 | 0.659 | 0.181 | 0.032 | 1.129 | 2.990 | 0.0114 | 0.819 |
| Mouse Subset Dataset | Lr_decay_epoch | | Default Lr_stage = 0.01 | | | | | | |
| | Basic_Loss | 1 | 0.694 | 0.197 | 0.063 | 0.966 | 2.170 | 0.014 | 0.820 |
| | | 10 | 0.672 | 0.186 | 0.072 | 1.111 | 2.270 | 0.0155 | 0.829 |
| | | 20 | 0.667 | 0.178 | 0.084 | 1.016 | 2.246 | 0.004 | 0.817 |
| | | 30 | 0.677 | 0.189 | 0.090 | 1.104 | 2.9656 | 0.014 | 0.823 |
| | Basic_MMD_Loss | 1 | 0.697 | 0.198 | 0.071 | 1.003 | 2.053 | 0.014 | 0.823 |
| | | 10 | 0.700 | 0.201 | 0.092 | 1.019 | 2.231 | 0.0131 | 0.834 |
| | | 20 | 0.668 | 0.200 | 0.082 | 1.016 | 2.204 | 0.014 | 0.823 |
| | | 30 | 0.667 | 0.199 | 0.084 | 1.110 | 2.230 | 0.011 | 0.821 |
