# Supplementary material for: Integration of unpaired single cell omics data by deep transfer graph convolutional network
Source: PLoS Comput Biol. 2025 Jan 16;21(1):e1012625. doi: 10.1371/journal.pcbi.1012625 (PMC11778791; doi:10.1371/journal.pcbi.1012625)
Supplement: S5 Table — (PDF) [file pcbi.1012625.s010.pdf]

S5 Tables. Performance results of view ablation study on Mouse Atlas Data.

	Abandon View		NMI	ARI	Silhouette	Purity	Davies-Bouldin Index	Jaccard Index	Integration ACC
Mouse Atlas Dataset	Lr_stage		Default Lr_decay_epoch = 10						
	Basic_Loss	0.01	0.803	0.397	0.132	1.032	3.193	0.015	0.650
		0.001	0.707	0.357	0.109	1.030	3.361	0.012	0.620
	Basic_MMD_Loss	0.01	0.803	0.411	0.165	1.095	2.93	0.026	0.696
		0.001	0.723	0.366	0.111	1.033	3.205	0.015	0.651
Mouse Atlas Dataset	Lr_decay_epoch		Default Lr_stage = 0.01						
	Basic_Loss	1	0.695	0.321	0.0891	0.944	3.463	0.008	0.596
		10	0.803	0.397	0.132	1.032	3.193	0.015	0650
		20	0.697	0.336	0.1326	1.034	3.396	0.012	0.620
	Basic_MMD_Loss	1	0.698	0.322	0.134	1.001	2.094	0.008	0.606
		10	0.803	0.411	0.165	1.095	2.93	0.026	0.696
		20	0.699	0.357	0.135	1.036	2.99	0.013	0.637
