# Supplementary figures and images for: Integration of unpaired single cell omics data by deep transfer graph convolutional network
Source: PLoS Comput Biol. 2025 Jan 16;21(1):e1012625. doi: 10.1371/journal.pcbi.1012625 (PMC11778791; doi:10.1371/journal.pcbi.1012625)

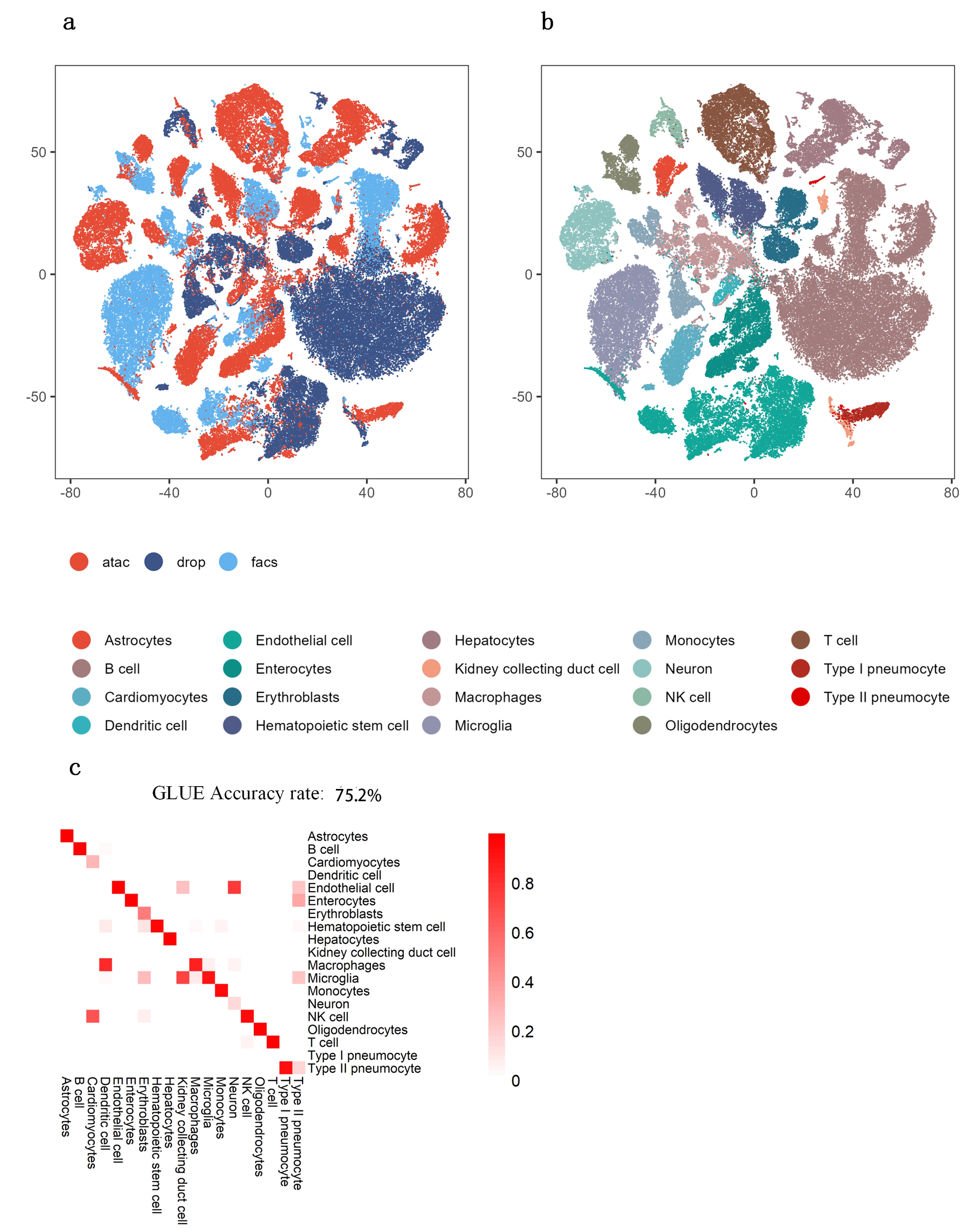

Supplement: S1 Fig — a, tSNE visualization of the overlapping subset data from mouse cell atlases for GLUE, colored by cell type. b, tSNE visualization of the overlapping subset data from mouse cell atlases for GLUE, colored by technology. c, Label transfer accuracy in overlapping subset data from mouse cell atlases. (TIF) [file pcbi.1012625.s011.tif]

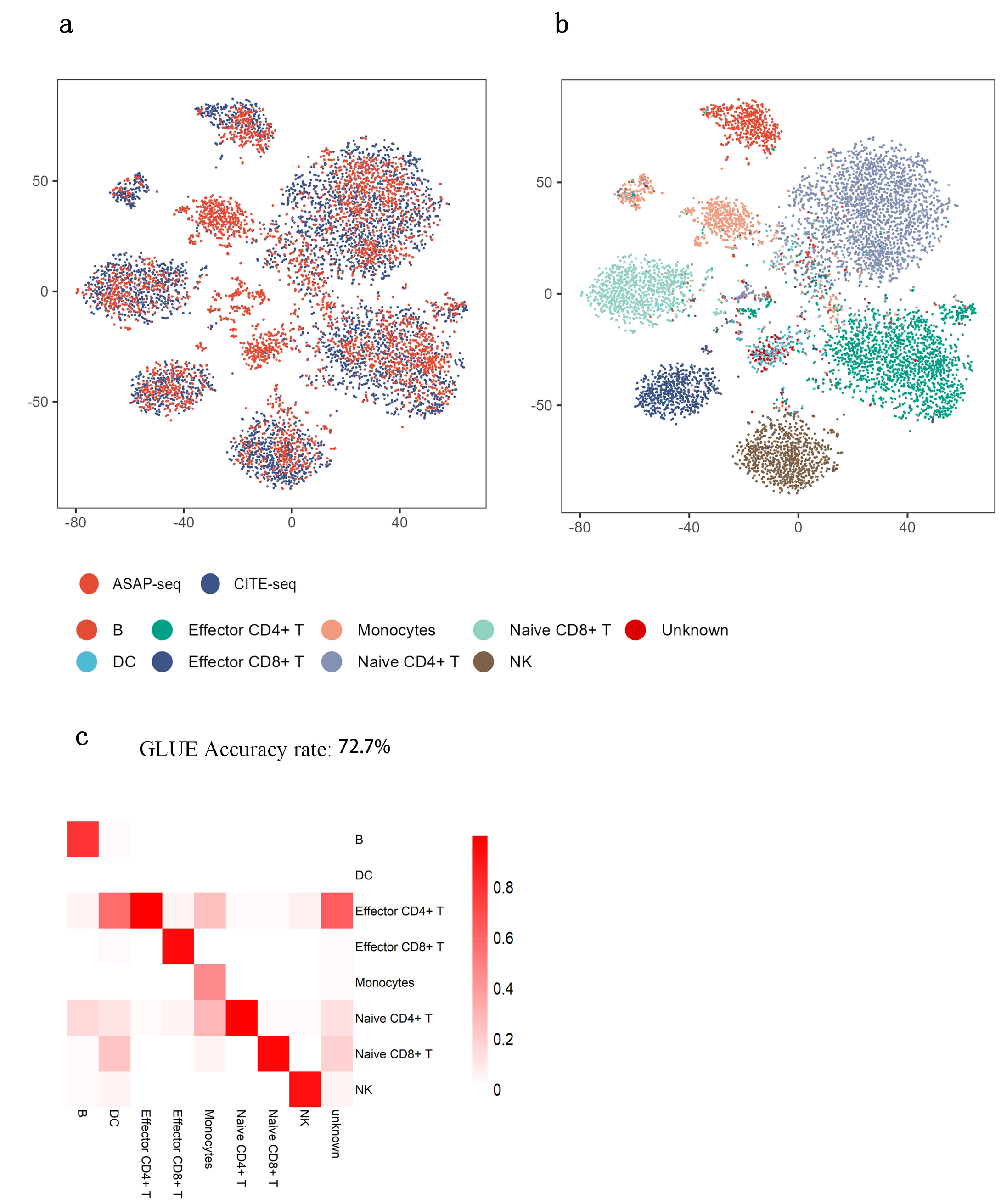

Supplement: S2 Fig — a, tSNE visualization of the PBMC data from mouse cell atlases for GLUE, colored by cell type. b, tSNE visualization of the overlapping subset data from PBMC data for GLUE, colored by technology. c, Label transfer accuracy in PBMC data. (TIF) [file pcbi.1012625.s012.tif]

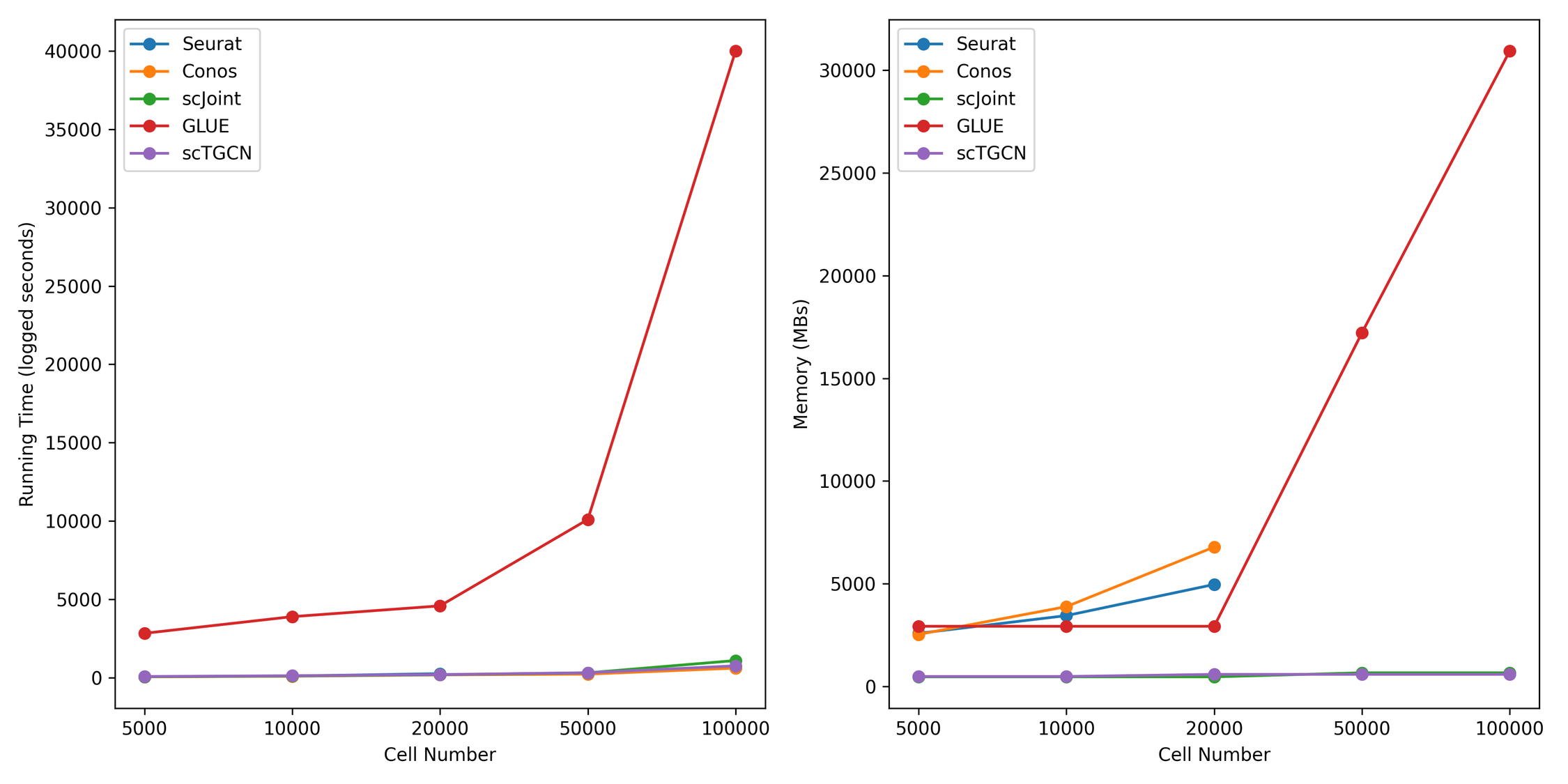

Supplement: S3 Fig — (TIF) [file pcbi.1012625.s013.tif]

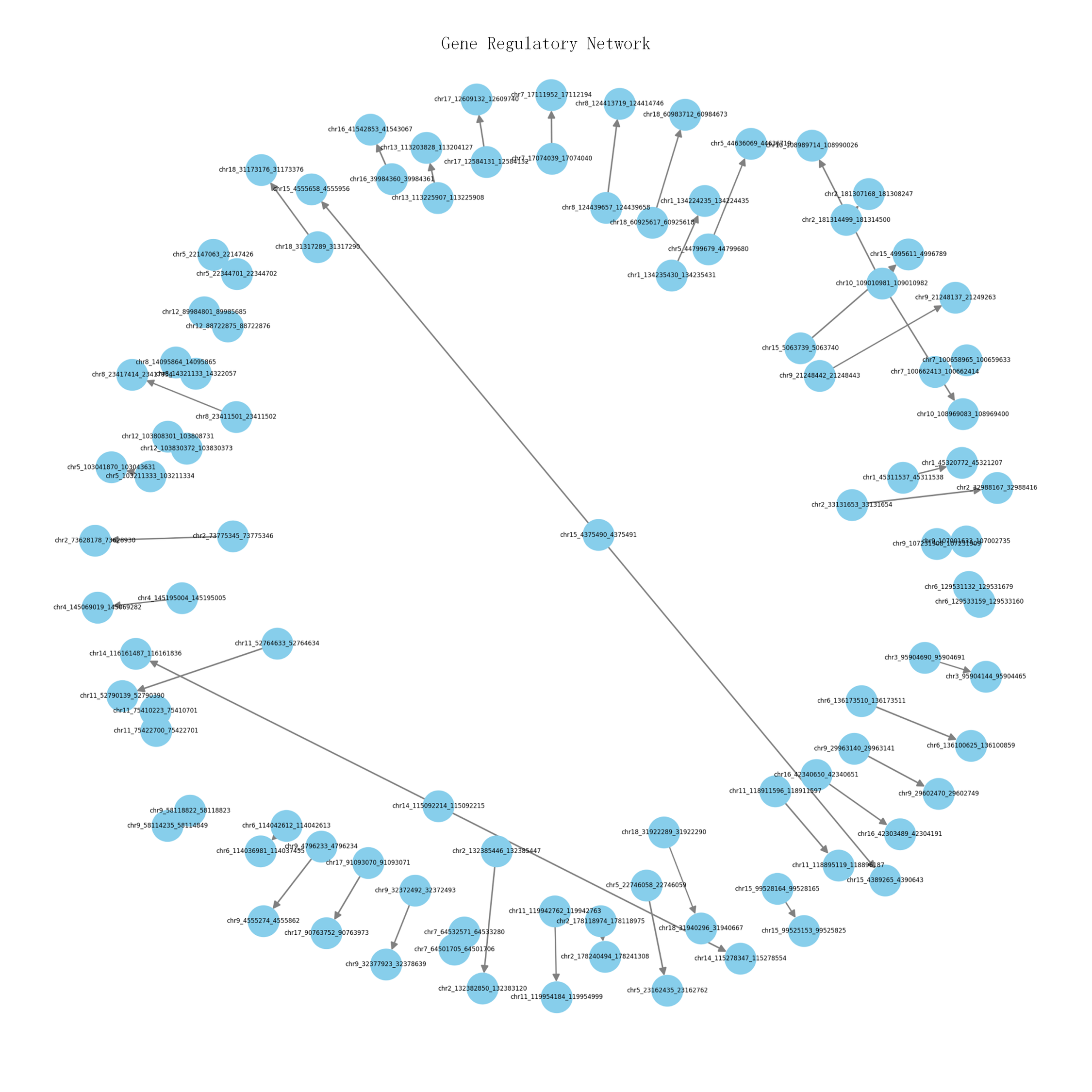

Supplement: S5 Fig — Some gene-peak relationship data is sampled from our gene2peak file. Each sampled line of data is split into seven values, including chromosome information, start and end positions for genes and peaks, and correlation values. Nodes representing genes and peaks are added to the graph G, and edges are created between genes and peaks with weights set to the correlation values. (TIF) [file pcbi.1012625.s015.tif]

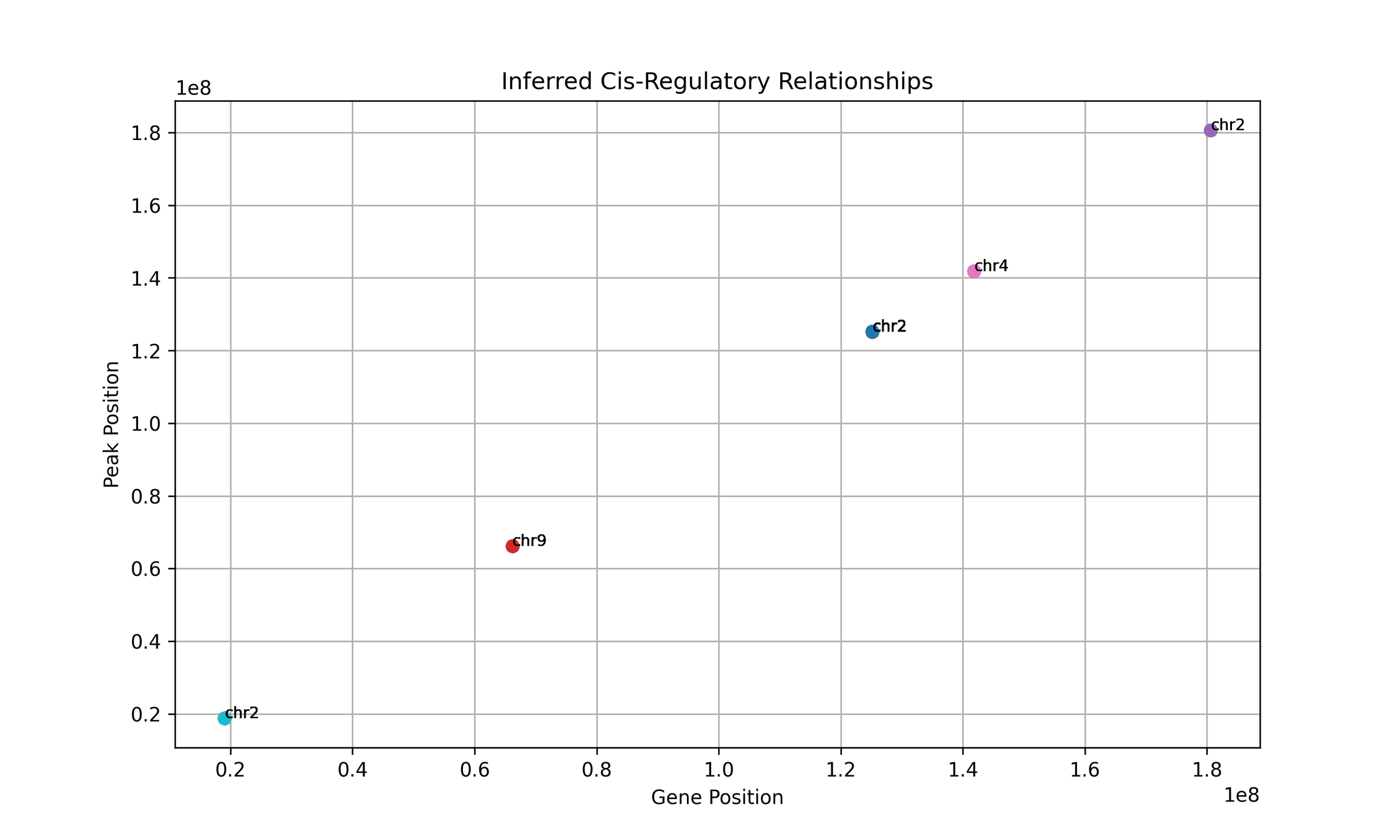

Supplement: S6 Fig — A line is drawn connecting the start and end positions of the gene and peak. A circular marker is placed at the peak position with a label showing the correlation score. The gene name is displayed near the circular marker for better identification. (TIF) [file pcbi.1012625.s016.tif]
